# Nerve fibers in the tumor microenvironment in neurotropic cancer—pancreatic cancer and cholangiocarcinoma

**DOI:** 10.1038/s41388-020-01578-4

**Published:** 2020-12-07

**Authors:** Xiuxiang Tan, Shivan Sivakumar, Jan Bednarsch, Georg Wiltberger, Jakob Nikolas Kather, Jan Niehues, Judith de Vos-Geelen, Liselot Valkenburg-van Iersel, Svetlana Kintsler, Anjali Roeth, Guangshan Hao, Sven Lang, Mariëlle E. Coolsen, Marcel den Dulk, Merel R. Aberle, Jarne Koolen, Nadine T. Gaisa, Steven W. M. Olde Damink, Ulf P. Neumann, Lara R. Heij

**Affiliations:** 1 Department of Surgery, Maastricht University Medical Centre, Maastricht, The Netherlands; 2 Department of General, Gastrointestinal, Hepatobiliary and Transplant Surgery, RWTH Aachen University Hospital, Aachen, Germany; 3 NUTRIM School of Nutrition and Translational Research in Metabolism, Maastricht University, Maastricht, The Netherlands; 4 Department of Oncology, University of Oxford, Oxford, UK; 5 Kennedy Institute of Rheumatology, University of Oxford, Oxford, UK; 6 Department of Medicine III, University Hospital RWTH Aachen, Aachen, Germany; 7 Division of Medical Oncology, Department of Internal Medicine, GROW School for Oncology and Development Biology, Maastricht University Medical Center, Maastricht, The Netherlands; 8 Institute of Pathology, University Hospital RWTH Aachen, Aachen, Germany; 9 Translational Neurosurgery and Neurobiology, University Hospital RWTH Aachen, Aachen, Germany

**Keywords:** Cancer microenvironment, Pancreatic cancer

## Abstract

Pancreatic ductal adenocarcinoma (PDAC) and cholangiocarcinoma (CCA) are both deadly cancers and they share many biological features besides their close anatomical location. One of the main histological features is neurotropism, which results in frequent perineural invasion. The underlying mechanism of cancer cells favoring growth by and through the nerve fibers is not fully understood. In this review, we provide knowledge of these cancers with frequent perineural invasion. We discuss nerve fiber crosstalk with the main different components of the tumor microenvironment (TME), the immune cells, and the fibroblasts. Also, we discuss the crosstalk between the nerve fibers and the cancer. We highlight the shared signaling pathways of the mechanisms behind perineural invasion in PDAC and CCA. Hereby we have focussed on signaling neurotransmitters and neuropeptides which may be a target for future therapies. Furthermore, we have summarized retrospective results of the previous literature about nerve fibers in PDAC and CCA patients. We provide our point of view in the potential for nerve fibers to be used as powerful biomarker for prognosis, as a tool to stratify patients for therapy or as a target in a (combination) therapy. Taking the presence of nerves into account can potentially change the field of personalized care in these neurotropic cancers.

## Introduction

Pancreatic ductal adenocarcinoma (PDAC) and cholangiocarcinoma (CCA) are aggressive cancers with only a limited response to chemotherapy. PDAC mortality is estimated to exceed the total breast, prostate, and colorectal cancer deaths and be the second leading cancer-related death by 2030 [1, 2]. PDAC and CCA share many clinical characteristics, which include high mortality rates and low treatment efficacy [3]. Unfortunately, survival rates have not improved even from recent novel therapeutic targets such as immune checkpoints [3–7]. Biologically PDAC and CCA are characterized by desmoplastic stroma and this stromal compartment is thought to be held responsible for the poor efficacy of chemotherapy. Today, surgery combined with chemotherapy is the only chance of cure [8].

The tumor microenvironment (TME) is a fervent area of research interest as it contains a host of nonmalignant cells that play an important role in carcinogenesis such as fibroblasts, immune cells, blood- and lymphatic vessels, and nerve fibers. In this review, we will focus on the pathways involved in neurogenesis and the interaction between nerve fibers and the other components of the TME.

Internal organs are innervated by the autonomic nervous system (ANS), which is composed of two components: the sympathetic nervous system (SNS) and the parasympathetic nervous system (PSNS). Increasing evidence shows that not only the internal organs are innervated by the PSNS, but solid tumors also depend on the development of nerves in the TME for growth and invasion in adjacent tissue [9–11].

**Fig. 1:**
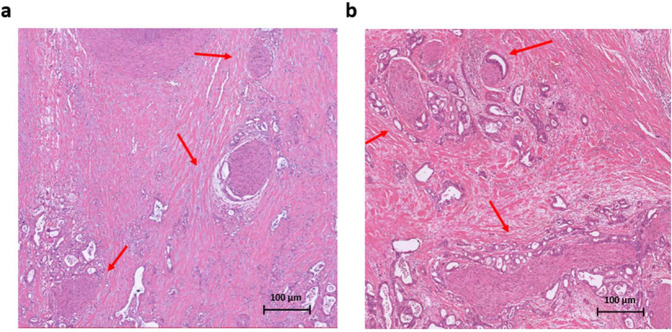
Histology of perineural invasion in neurotropic cancer. Histology slide of PDAC (**a**) and CCA (**b**) in Hematoxylin and Eosin (H&E) showing PNI, which is one of the shared pathological characteristics of both cancers. **a** PDAC slide with extended PNI and an almost identical histomorphology as CCA. **b** CCA with tumor cells massively invading the perineural space, surrounded by desmoplastic stroma and few small tumor glands in the stroma.

Besides the aggressive behavior and poor response to treatment, another shared feature of these two cancer types is perineural invasion (PNI), which is defined as cancer cells surrounding at least 33% of the epineurial, perineural, and endoneurial space of the nerve sheath [12]. PNI describes the process of cancer cells invading the nerve, crossing all layers of the nerve sheath. Once the cancer cells are invaded in the nerve, they have reached a favorable environment to travel intraneural and contribute to the progression of the disease. Over time different definitions for PNI have been used. It has been described as cancer cells located in the endoneurium associated with the Schwann cells [13] or later on as the presence of cancer cells along one of the layers of the nerve sheath [12, 14–16]. For pathologists invasion in one of these nerve sheath layers is used to report PNI and often a mixture of invasion in different layers is seen in one histological slide. Intraneural invasion in PDAC has been associated with higher frequency of local/distant recurrence when compared to cases with PNI but without intraneural invasion [17]. This provides some evidence that defining the level of PNI matters clinically but up to now this is not recommended in the guidelines for the pathologists. The exact underlying mechanism of PNI remains unknown [12, 18]. A hypothesis is that the nerve fibers choose the path of “least resistance” and the cancer cells move along this low resistance path [14, 15]. A recent insight showed that PNI was activating signaling pathways when cancer cells attacked the perineural spaces of the surrounding nerves [12]. Even though PNI commonly occurs in many solid tumors [19–22], PDAC and CCA are “neurotropic cancers” and have a high frequency of PNI [23]. It has been reported that almost all PDAC lesions contain PNI and about 75% of CCA lesions showed PNI [23–25] (Fig. 1 presents the classical PNI pathological characteristics of PDAC and CCA).

A novel biological phenomenon is the cancer-related neurogenesis, which is described in prostate cancer. The nerve fiber density is increased in paraneoplastic and neoplastic prostate lesions [26]. It is not known whether this cancer-related neurogenesis also occurs in PDAC or CCA. Exploring the role of alterations in nerve fibers in PDAC and CCA has the potential to be of importance in developing personalized medicine and finding an effective novel treatment strategy.

In this review, we aim to provide an overview of the current knowledge about nerve fiber crosstalk with cancer, and other components of the TME in PDAC and CCA.

## Innervation and neurotransmitters

**Fig. 2:**
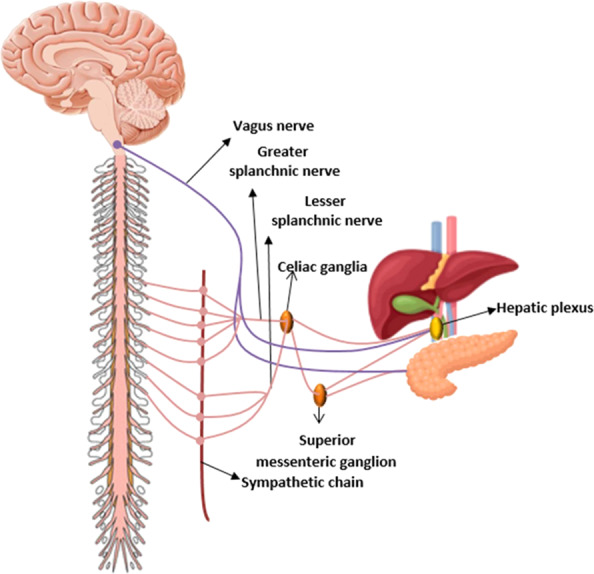
A schematic overview of normal pancreas and liver innervation by the PSNS (in purple) and the SNS (in pink). SNS innervation of the liver and pancreas is derived from the lateral horn of the spinal cord (liverT7-T12, pancreas C8-L3) across through sympathetic chain primarily via the splanchnic nerve to prevertebral ganglia including the celia and superior ganglion entering hepatic plexus. The PSNS supporting the liver and pancreas originates from the brainstem (dorsal motor nucleus of the n. vagus in the medulla) and activates parasympathetic postganglionic neurons in the liver or the pancreatic ganglia [28–31].

There is a complex nerve fiber network distributed around the pancreas, retroperitoneum and the biliary tree. Nerve fibers in the PDAC TME include axons originating from the sympathetic, parasympathetic, enteropancreatic or hepatic plexus, afferent nerve fibers and newly developed nerve fibers [27]. The nerve fibers of the SNS are derived from the lateral horn of the spinal cord whilst PSNS fibers originate from the brainstem (Fig. 2) [28–31]. A rich nerve network facilitates peripheral nerve invasion by cancer cells. Direct contact between cancer cells and nerves leads to a fertile ground for PNI. In addition, numerous signal transduction pathways, neurotransmitters, and neuropeptides regulate the pathophysiology of cancer cells [32–34]. Neurotransmitters can interact with cancer cells and in exchange the cancer cells can also release neurotransmitters which can activate receptors on nerve fibers. This stimulation possibly causes dysregulation of the nerve fiber network and acceleration of PNI [35].

### Norepinephrine (NE)

NE is an indispensable neurotransmitter released by the postganglionic sympathetic neurons that is involved in cell migration activity [36]. The migration of tumor cells is needed for the development of cancer metastases [37]. Migration activity mediated by norepinephrine occurs via acting on β-2 adrenergic receptors and this can be influenced by using β-blockers [38]. A study in mice demonstrated that when NE is induced in development, the secretion of neurotrophins is accelerated. The inhibition of neurotrophins receptors improved the effect of gemcitabine’s treatment (gemcitabine is a chemotherapy commonly used in pancreatic cancer) [39]. In CCA cell lines (Mz‐ChA‐1 cells), using immunocytochemistry and immunoblotting α-2‐adrenergic receptor was stimulated by NE, causing an upregulation of cAMP. cAMP stimulates or inhibits mitosis depending on the cell type. By prolonged EGF stimulation, by NE or cAMP, an increase of RAF-1 and B-RAF was achieved. This opens new possible therapeutic targets because the inhibition of growth occurred downstream of RAS [40]. The study demonstrated that β-2 and α-2 adrenergic receptors were significantly downregulated in a differentiation signature involved in the above process and promote cancer progression. Thereby, p53 mutation was linked to poor survival. Deficiency of p53 can lead to cancer-associated neurogenesis with an adrenergic phenotype and a poor survival in head and neck cancer [41].

### Acetylcholine (Ach)

Ach and its ligands nicotinic acetylcholine receptors (nAChRs) participate as functional neurotransmitters in the cholinergic system and they play a stimulating role in the progression of PDAC and CCA [42–44]. In CD18/HPAF pancreatic cell implantation in mice, nicotine treatment stimulated the α7 subunit of nicotinic acetylcholine receptor (7-nAChR) and enhanced cancer metastasis. This stimulation of 7-nAChR resulted in an activation of Janus kinase2 (JAK2)/STAT3 signaling cascade together with the protein kinase (Ras/Raf/MEK/ERK1/2) pathway [45]. Also, higher densities of muscarinic Ach receptor 3 (M3) showed an association with high-grade differentiation of PDAC, more lymph node metastasis and a shorter patient overall survival (OS) [44]. In CCA, the presence of the cholinergic system plays a role in CCA cell proliferation and growth [46, 47].

### Nerve growth factor (NGF)

NGF has been extensively studied and it has been shown that NGF not only acts directly on the peripheral and central nervous system, but also on the components of the TME [48]. NGF treatment can trigger the cancer cells to stay in a more differentiated cell phenotype and thus reduce tumor growth [48, 49]. Besides the effect on the cancer cell phenotype there is a possible interaction between the immune cells in the TME. NGF is able to act on immune cell activities and this enables NGF to play an important role in the immunity against cancer. Two important cell surface receptors on neurotrophins have been described: tropomyosin receptor kinase A (TRKA) and p75 neurotrophin receptor [50–52]. In CCA patients, high levels of NGF and high levels of TRKA have been shown to be a marker for poor prognosis [53]. In PDAC, overexpression of NGF promoted pancreatic cell proliferation and invasiveness and was associated with poor prognosis of PDAC [54]. Saloman et al. injected NGF antibodies in a xenograft mouse model of PDAC. It appeared that anti-NGF treatment reduced neural inflammation, neural invasion, and metastasis in this model only after recent onset of the disease. This indicates that the timing of treatment would be critical [55]. Furthermore in the biliary system in rats it was shown that cholangiocytes secrete NGF and express NGF receptors. NGF promoted cholangiocyte proliferation in synergy with estrogen [56]. It has been shown in human CCA cell lines (QBC939) that NGF-β induced progression of the disease [57]. Other studies also confirmed that the role of NGF-β in human hilar CCA was associated with lymph node metastasis and nerve infiltration [58].

## Crosstalk between neural systems and cancer

**Fig. 3:**
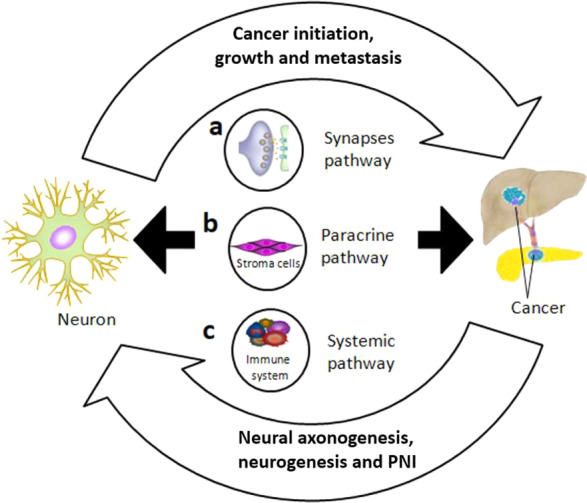
A bidirectional crosstalk between nerves and cancer in pancreas and liver. Nerve fibers as branches of neuron infiltrate the TME and control cancer cells initiation, growth and metastasis mainly through three interactions pathways: **a** Direct contact in a synapse pathway, neuron as presynaptic cell via secretion of neurotransmitters and neuropeptides such as acetylcholin act on neurotransmitter receptors such as acetylcholin receptors on postsynaptic cell to regulate epithelial proliferation, stem cell activity, both nerve and liver or pancreatic cancer cells can be as pre- or post-synaptic cells in this communication. **b** Paracrine pathway, within TME, addition to direct influence, nerves communication with cancer can be mediated by regulation of tumor-related stroma cells. And the paracrine signals (neurotrophic growth factors) derived by cancer cell promote nerve axonogenesis or neurogenesis in TME. **c** Systemic pathway, nervous system can influence cancer cells activities through regulation the function of immune system mediated by elevated systemic circulating catecholamines. PNI can also be a consequence of circulating signals from cancer cells [42, 59].

Two recent reviews reveal the importance of nerves in cancer and describe the bidirectional crosstalk of the nervous system. The nerves are able to control cancer initiation, growth and metastasis, whereas the cancer induces functional alterations of the nervous system (Fig. 3 shows an overview of the bidirectional crosstalk) [49, 59]. Nerves often travel together with blood vessels, mainly arterioles and capillaries, and share the same distribution [60]. In addition, adrenergic nerves can activate and initiate angiogenesis in the early stage of cancer [9]. For the tissue to make the transition from hyperplasia to neoplasia there is a need for angiogenic activity and neovascularization [61]. Evidence showed that adrenergic nerves, through the ß-adrenergic receptor pathway, are important in facilitating tumor growth and cancer cell dissemination in prostate cancer [62, 63]. Hence pharmacological blockade of adrenergic-ß-blockers can improve survival [62]. Previous work suggests that a ß-blocker therapy could show lower recurrence and lower mortality in many types of cancer including PDAC [63–66]. Nerves contribute to the development of a new vascular network and they help in supplying the TME for oxygen, nutrients, and removal of waste products [9]. Neurogenesis is driven by neural progenitors in the TME which are strongly associated with tumor infiltration and dissemination [67]. An increased density of nerve fibers in the tumor lesion is correlated with high-grade disease and increased pathological stage, including for cancers of head and neck [68], breast [69], lung [70], stomach [71], prostate [26, 62], colon and rectum [72]. For PDAC it is suggested to be the other way around: a low nerve fiber density correlates with a poorer survival [73]. In another study, a high nerve fiber density of the parasympathetic nerve fibers correlated with tumor budding and a poor survival [74].

Nerve fibers from different origins have a different influence and can be indicative of a protective or aggressive role. Previous work has investigated the role of adrenergic nerves in prostate cancer, this work illustrates that the adrenergic nerve fibers are important in the “angiogenic switch”, which activates endothelial cells to support exponential tumor growth [9]. Analysis of clinical outcome in 43 prostate cancer patients showed that parasympathetic nerve fibers are involved in early tumor development and the parasympathetic nerve fibers play a role in the tumor progression and more disseminated disease [62]. A retrospective study of PDAC specimens found consistent results and in PDAC the parasympathetic neurogenesis is highly associated with a poor prognosis [74]. PDAC mouse model experiments have investigated the role of nerves in the TME, multiple studies have shown that targeting the nerves in the TME could contribute to the prevention of paraneoplastic lesions to develop into cancer [75–77].

In one of these experiments, xenograft mouse models and genetically engineered mouse models of PDAC are used to investigate the role of peripheral nerves in the paraneoplastic lesion, where neonatal capsaicin is administered to ablate the sensory innervation of the pancreas. The results show that in the early stage of cancer, denervation of the pancreas slows pancreatic intraepithelial neoplasia progression and ultimately increases survival [76]. Denervation treatment has been shown to be effective in early stages of cancer. However, in a metastatic PDAC mouse model, mice with vagotomy (with a part of the PSNS) have an accelerated tumorigenesis. Systemic administration of a muscarinic agonist reduced this effect in the mice that underwent vagotomy [7]. These results indicate that denervation treatment combined with another therapy can be effective.

During the tumor formation process, tumor cells may attract neural progenitors which induce neurogenesis to support their growth and metastasis. Adrenergic nerve fibers and newly formed neural networks develop and infiltrate into the cancer-related stroma, providing signals to regulate tumor progression [78]. Previous studies have presented that newly formed adrenergic nerve fibers are participators involved in prostate carcinogenesis and progression and this was associated with a poor clinical outcome [9, 26, 62]. Recently it was shown that p53 mutation status in head and neck cancer was related with an adrenergic transdifferentiating of the nerves in the tumor and this was associated with a poor clinical outcome [78].

## Crosstalk between nerves and immune cells

Previous literature shows that communication and interaction between the nervous system and the immune system exists. The ANS has a regulatory effect on the inflammatory response [79]. The peripheral nervous system interacts by efferent and afferent nerves which innervate primary and secondary lymphoid organs such as the spleen and lymph nodes. T cells originate in the thymus and then spread to peripheral organs [80]. During the process of T cell development and differentiation, neuro-immune communications in the thymus play a key role [81]. A current study reveals the distribution of nerve fibers in mouse thymus venules: there is a dense network of nerve fibers present in all thymic compartments. Inside the thymus, nerve fibers are closely associated with the blood vessels including postcapillary venules. This indicates that neural regulation may be involved in lymphocyte transport since T cell precursors and mature lymphocytes enter the peripheral organs through postcapillary venules [82]. In our own recent study, we show in our cohort of PDAC patients, that nerve fibers are co-localized with clusters of lymphocytes. These clusters are mainly CD20 positive B cells, CD4+ CD8+ T cells and CD21+ follicular dendritic cells (unpublished data).

Besides the co-localization of nerve fibers with immune cells, the patients with a high nerve fiber density show a better survival. This phenomenon is described as neuro-immune cell units (NICUs), in other studies: immune cell and nerve fiber co-localization and their interaction can drive tissue protection and can play a critical immune-regulatory role [83, 84]. NICUs are present in many tissues around the body and are shown to be important in many physiological processes such as tissue repair, inflammation and organogenesis [85, 86]. However, the current knowledge of NICUs is limited. In conclusion, exploring this prominent novel field in cancer research will unravel future pathways for a better response to novel therapy strategies and may make PDAC and CCA patients more primed for a better response to immunotherapy.

## Crosstalk between nerves and fibroblasts

Within the TME, cancer-associated fibroblasts (CAFs) are the major stromal cell type (up to 80% of the tumor mass in PDAC). The remodeling of the stromal compartment contributes to cancer growth and progression by activation of secretion of cytokines [87, 88]. PDAC and CCA are typically characterized by a significant desmoplastic reaction. The desmoplastic stroma creates a physical and chemical barrier to prevent therapeutics and immune cells to infiltrate and reach the cancer. This results in an immune cell depleted TME and could be an explanation for failure of response to immunotherapy [89]. In addition, during the ECM remodeling, a type I collagen predominate phenotype, tends to stimulate angiogenesis and neurogenesis facilitating neo-vessel formation, which is beneficial for tumor dissemination [49]. In PDAC and CCA, CAFs are differentiated from stellate cells, these stellate cells play an essential role in the desmoplastic reaction through the expression of alpha-smooth muscle actin (α -SMA) and co-localization with procollagen, contributing to ECM remodeling [3]. Furthermore, stellate cells favor nerve outgrowth during tumor development by supporting growth and elongation of neurons and communicate via Ach signaling pathways [90]. A few studies have described that hepatic stellate cells regulate nerve growth via the alteration of ECM composition and up-expression of several factors such as tumor growth factor beta, which stimulates angiogenesis and with this influences axons in the TME [91]. Abundant ECM components such as hyaluronic acid, fibronectin and collagen are predominately present within the TME in PDAC and CCA. Those ECM components influence neuron growth [92, 93]. Hence, stellate cells regulate the composition of ECM components and therefore affect nerve growth in PDAC and CCA [90].

CAFs can secrete matrix metalloproteases (MMPs), the MMP family is an ECM protein and they are reported to be regulators of neural development [94]. It was described that some MMPs (MT1-MMP) are shown to degrade the ECM and promote pancreatic cancer expansion, invasion, and progression to an advanced stage [95, 96]. It is reported that MMP9 is associated with more lymph node metastasis and a poorer survival in breast cancer [97]. PDAC cells in vivo undergoing chronic stress, were sensitive to neural signaling and pancreatic stromal cells were increased. This promoted tumor metastasis and cancer progression, β-adrenergic receptor blockade intervention therapy can block this neural signaling and be part of a combination therapy for PDAC [63].

## Pathological features of nerve fibers in PDAC and CCA

PNI is considered as an important factor for poor prognosis in PDAC and CCA [24, 98]. It has been shown that PNI can be the reason for curative resection failure (shown in Table 1). Chatterjee et al. showed by examination of 212 PDAC slides, that the presence of PNI was directly correlated with tumor size, margin status, lymph node metastasis, and AJCC stages and inversely associated with disease free survival (DFS) and OS [17]. Shimada et al. found that the degree of intrapancreatic nerve invasion can be used as a predictor for recurrence of disease after surgery [99]. A phenomenon termed as “neural remodeling” is postulated in PDAC, characterized by the alterations in morphology of the nerve [18, 100–102]. It was shown that nerve fiber alterations including hypertrophic nerves, increased nerve fiber density and pancreatic neuritis were strongly associated with GAP-43 overexpression and abdominal pain [103]. In the perineural space, PNI induces reactive alterations in the morphology and function of the nerves. Morphological changes include changes of the nerve trunk and thickness [102]. The aggressiveness of PNI is related to neural remodeling, desmoplasia and cancer pain. Severe and enduring pain was strongly associated with poor survival in PDAC patients. However, these neural alterations did not have a significant association with survival [103]. NGF and Artemin play a fundamental role in neural modeling in pancreatic adenocarcinoma.

**Table 1:** Summary of current research neural remodeling in PDAC and CCA.

References	Samples	Neural marker	Key results	Cancer types
Iwasaki et al. [73]	256	GAP-43	Neural hypertrophy and nerve numbers are decreased;	Pancreatic carcinoma
Fisher et al. [105]	58		Presence of PNI was associated with a reduction of OS	Cholangiocarcinoma
Chatterjee et al. [17]	212		Presence of PNI was directly correlated with tumor size, margin status, lymph node metastasis, pathologic tumor and AJCC stages, and shorter DFS and OS	Pancreatic carcinoma
Lenz et al. [106]	177		80.3% of PDAC detected PNI; Neural remodeling progress can be a reason of pancreatic neuropathy;	Pancreatic carcinoma
Shimada et al. [99]	153		Degree of PNI was directly related to lymph node metastases, surgical margin and tumor sides size, and inversely associated with DFS	Pancreatic carcinoma
Ceyhan et al. [107]	97	GAP-43 NGF PGP9.5 Artemin	There were enlarged nerves and dense neural networks in pancreatic carcinoma slides; Neurotrophic characteristics GAP-43, NGF and Artemin are increased in pancreatic carcinoma and have close relevancy with intrapancreatic neuropathy.	Pancreatic carcinoma
Ceyhan et al. [103]	149	GAP-43 PGP9.5 NGF	Neural hypertrophy, neural density increased; Neuropathic alteration was not correlated with survival in pancreatic carcinoma, these changes include neural thickness, hypertrophy, density. Only the severity of pain significantly affects survival.	Pancreatic carcinoma
Ceyhan et al. [103]	564	GAP-43	Neural density is increased and nerves are enlarged in pancreatic carcinoma, the severity of PNI was linked to neuropathic changes and pain.	Pancreatic carcinoma
Shirai et al. [104]	59		80% of CCA samples detected PNI; 5 years survival rates of patients with or without PNI were 17% and 70%, respectively.	Cholangiocarcinoma

Interestingly, lower intrapancreatic neural density in the tumor area was linked to shorter OS with multivariate analysis [73]. Related research in CCA mainly focused on PNI prevalence and patient survival [45, 99, 104]. In summary, these studies consistently showed that the presence of PNI was linked to shorter survival in patients with PDAC or CCA. The significance that PNI is an independent prognostic factor of poor outcome has been demonstrated. However, the influence of nerve remodeling especially the new outgrown small nerve fibers has not been fully explained.

## Conclusions and future directions

With its frequent PNI, PDAC and CCA are two neurotropic cancers. The neurotropism of these cancers could be an explanation of their aggressiveness and poor response to treatment. In this review, the progress of recent research in the mechanism of PNI in PDAC and CCA is discussed. However, the crosstalk between the nervous system in PDAC and CCA is undiscovered. Different nerve fibers have a different function and the interaction with the components of the TME and the cancer are important to investigate.

In PDAC, the role of nerve fibers is divergent and nerves from the PSNS and SNS have a cancer stimulating and cancer inhibiting effect. To our current knowledge, detailed understanding of the underlying mechanisms of tumor and nerve fiber interaction is critical for the development of innovative therapeutic strategies for patients with these highly lethal cancers. The nerve outgrowth is part of the TME, in which cancer to stroma crosstalk takes place. It is likely that other components of the TME also influence the nerve outgrowth and immune cells and fibroblasts are key components in this process. Targeting nerves has the potential to be a new strategy for therapy for PDAC and CCA patients by influencing the TME, immune cells and fibroblasts, potentially influencing sensitivity to therapeutics. The newly formed nerve fibers are different from the more commonly used PNI. From our perspective, PNI originates in the pre-existing nerve fiber networks and the cancer uses the distribution network for cancer growth. This is a well-known sign of aggressive disease and is associated with poor survival. Small nerve fiber outgrowth can be used as a biomarker for a better survival, as a tool to stratify patients for treatment and as a target for therapeutics. More research is needed to investigate whether sensitivity to already existing immunotherapy can be achieved by targeting nerves.
